# Biophysiochemical properties of endothelial cells cultured on bio-inspired collagen films

**DOI:** 10.1186/1472-6750-14-61

**Published:** 2014-07-02

**Authors:** Eunseok Seo, Kyung Won Seo, Jung-Eun Gil, Young-Ran Ha, Eunseop Yeom, Seungchul Lee, Sang Joon Lee

**Affiliations:** 1 Division of Integrative Biosciences and Biotechnology, Pohang University of Science and Technology, San 31, Hyoja-dong, Nam-Gu, Pohang, Gyeongbuk 790-784, Korea; 2 Center for Biofluid and Biomimic Research, Department of Mechanical Engineering, Pohang University of Science and Technology, San 31, Hyoja-dong, Nam-Gu, Pohang, Gyeongbuk 790-784, South Korea; 3 Department of Biological Sciences, Center for Cognition and Sociality, Institute for Basic Science, 291 Daehak-ro, Yuseong-gu, Daejeon 305-701, South Korea

## Abstract

**Background:**

In this study, we investigated the effect of the extracellular matrix on endothelial dysfunction by careful observation of human umbilical vein endothelial cells (HUVECs) cultured on denatured collagen film.

**Results:**

HUVECs on denatured collagen film showed relatively high surface roughness compared with normal HUVECs. The expression levels of MMP-1, MMP-2 and CD146 increased in the ECs on denatured collagen film. In addition, we examined the accumulation of fluorescent beads on HUVEC layers subjected to circulatory flow. The number of accumulated fluorescent beads increased on the disorganized HUVEC layers.

**Conclusions:**

The proposed *in vitro* study using bio-inspired collagen films could potentially be used in the size- and ligand-based design of drugs to treat endothelial dysfunction caused by circulatory vascular diseases.

## Background

Atherosclerosis is a major cause of morbidity and mortality [1]. Many *in vitro* experiments on the early development stage of atherogenesis have been performed using pro-inflammatory mediators, such as lipopolysaccharide in bacterial cell walls [2-9], as stimuli. Endothelial cell (EC)-leukocyte interaction of these stimuli induces changes in shape, permeability, and gene expression of ECs [10-13]. However, previous studies overlooked the effects of the extracellular matrix (ECM) on variations in morphology and growth of ECs. Thus, this study experimentally investigated the effect of ECM properties on endothelial dysfunction.

The EC layer functions as an interface between circulating blood and surrounding tissues [14,15]. ECs modulate thrombosis and inflammation, and control mural smooth muscle cells and vascular health. These cells also function in physiological processes, such as innate and adaptive immune responses. Thus, endothelial dysfunction is associated with the development of atherosclerosis and other cardiovascular disorders [16-18]. ECM degradation, which is possibly regulated by matrix-degrading metalloproteinases (MMPs) and their endogenous tissue inhibitors, is closely involved in the outbreak of atherogenesis [19-21]. MMPs belong to a family of zinc metalloendopeptidases. These enzymes, which degrade ECM proteins, exhibit important functions in embryo development, morphogenesis, and tissue remodeling, as well as in various diseases, such as arthritis, atherosclerosis, invasion, and metastasis of cancer cells [22]. A maximum of 25 MMP members are found in humans and animals. Among these MMPs, 14 are related to atherosclerosis [23]. Vascular cells, including ECs and macrophages, secrete MMPs. ECs also express MMP-1 (collagenase), MMP-2 (gelatinase), and MMP-3 (stromelysin) [24-26].

The mechanical properties of arterial walls and susceptibility to pathological vascular remodeling are mainly determined by the macromolecular structures of collagen, elastin, and proteoglycans [27-29]. Under low pressure, the high compliance of elastin dominates the wall displacement. At high pressure, the wall displacement is limited by the stiffer collagen. Degradation of these mechanical properties with aging [30], smoking [31], diabetes [32], hypertension [33], and atherosclerosis [29,34] is associated with changes in the collagen and elastin scaffold. Although these macromolecules generate the biphasic mechanical response of arterial walls to pressure, the specific features of their microstructure remain as a missing element in the modeling of wall mechanics [35,36]. Therefore, we hypothesized that the structural change in collagen by MMP-1 and MMP-2 expression may induce endothelial dysfunction, such as disorganization and thickening of the EC layer. To validate this hypothesis, we prepared two different collagen films as ECM models. The normal collagen film corresponded to the ECM of normal blood vessels, and the denatured collagen film was adopted to mimic the ECM of atherosclerotic blood vessels. The denatured collagen film was intentionally disrupted by collagenase treatment to simulate the denatured ECM by increasing MMP-1 and MMP-2 expression under *in vivo* conditions. Through preliminary experiments using zebrafish models for investigating the early development stage of atherogenesis, we observed an EC monolayer surrounded by a collagen basement membrane in the normal vascular endothelium. The vascular endothelium of the early development stage of atherogenesis shows the disorganization and thickening of the EC layer [37]. To simulate these morphological results, we cultured human umbilical vein endothelial cells (HUVECs) on two different types of collagen films.

In this study, morphological variations in the bio-inspired ECM were experimentally investigated. The spatial distribution of surface roughness of ECs on the ECM was observed by phase-contrast digital holographic microscopy (DHM). Biological specimens, such as living cells, are transparent. However, transparent biological samples are difficult to observe clearly by bright-field microscopy. Therefore, non-invasive high-resolution imaging of living cells under *in vivo* conditions is important to visualize biological processes. Interferometry-based DHM can be performed to determine the spatial distributions of the phase and optical path length of a test sample. This technique can also provide quantitative phase information of a sample with a spatial resolution of tens of nanometers [38-41]. Temporal variations in morphological structure and EC layer thickness can be clearly observed with a time-resolved DHM technique.

To study the mechanism of endothelial dysfunction, we investigated the expression of CD146, vascular cell adhesion molecule (VCAM), and E-selectin on the surface of abnormal ECs. These three molecules are used as biomarkers of EC injury [6,17,42-45]. We also examined the positional information of vascular endothelial cadherin (VE-cadherin) of ECs. VE-cadherin has an important function in controlling vascular organization and modulating endothelial permeability [46].

To develop an optimal delivery system for vascular targeting and maximize drug accumulation on the EC layer while avoiding entrapment in the lung and small capillaries [47], the specific ligand-receptor interaction with the vessel walls [48] and surface properties related to specific vascular adhesion and reduced macrophage uptake should be investigated. To establish an *in vitro* study for drug screening of circulatory diseases, fluorescent bead accumulation on EC layers was examined under circulating flow conditions.

## Results

### Collagen film and denatured collagen film

**Figure 1:**
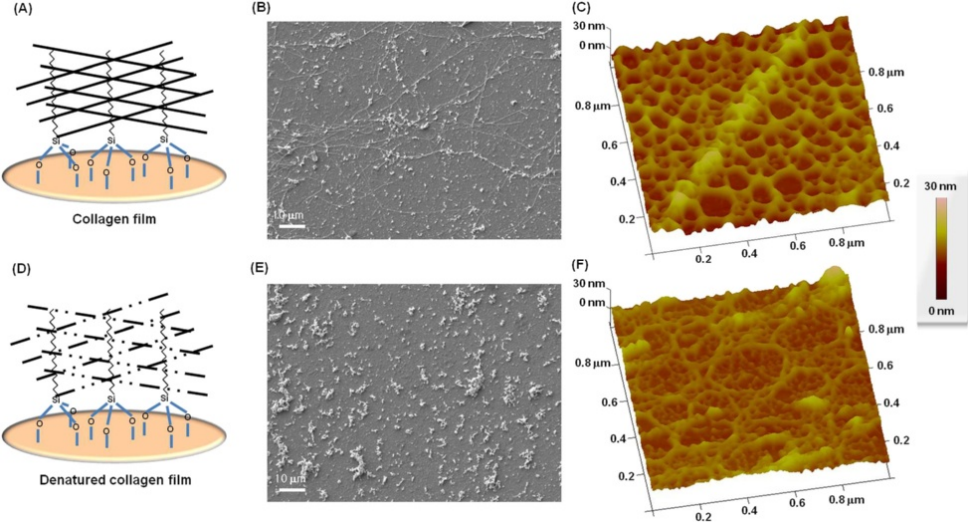
**Two collagen films prepared for the *in vitro *study. (A)** Normal collagen film, which mimics the ECM of normal blood vessels. Typical SEM image **(B)** and AFM image **(C)** of the collagen film. **(D)** Denatured collagen film, which mimics the ECM of abnormal blood vessels. The structure of the denatured collagen film was intentionally broken using collagenase to simulate the abnormal ECM. Typical SEM image **(E)** and AFM image **(F)** of the denatured collagen film.

Figures 1A and 1D illustrate the collagen and denatured collagen films coated on octadecyltrichlorosilane (OTS)-self-assembled monolayer (SAM)-treated coverslips. Figures 1B and 1E show typical scanning electron microscopy (SEM) images (1000×) of HUVECs on the collagen and denatured collagen films, respectively. The surface structures of the two films were also observed using atomic force microscopy (AFM). Figures 1C and 1F are typical AFM images showing the morphological structures of the collagen and denatured collagen films, respectively. As shown in Figures 1B and 1C, the collagen film had fiber structures. However, the fibers were broken in the collagenase-treated collagen film (Figures 1E and 1F).

### Roughness of ECs

**Figure 2:**
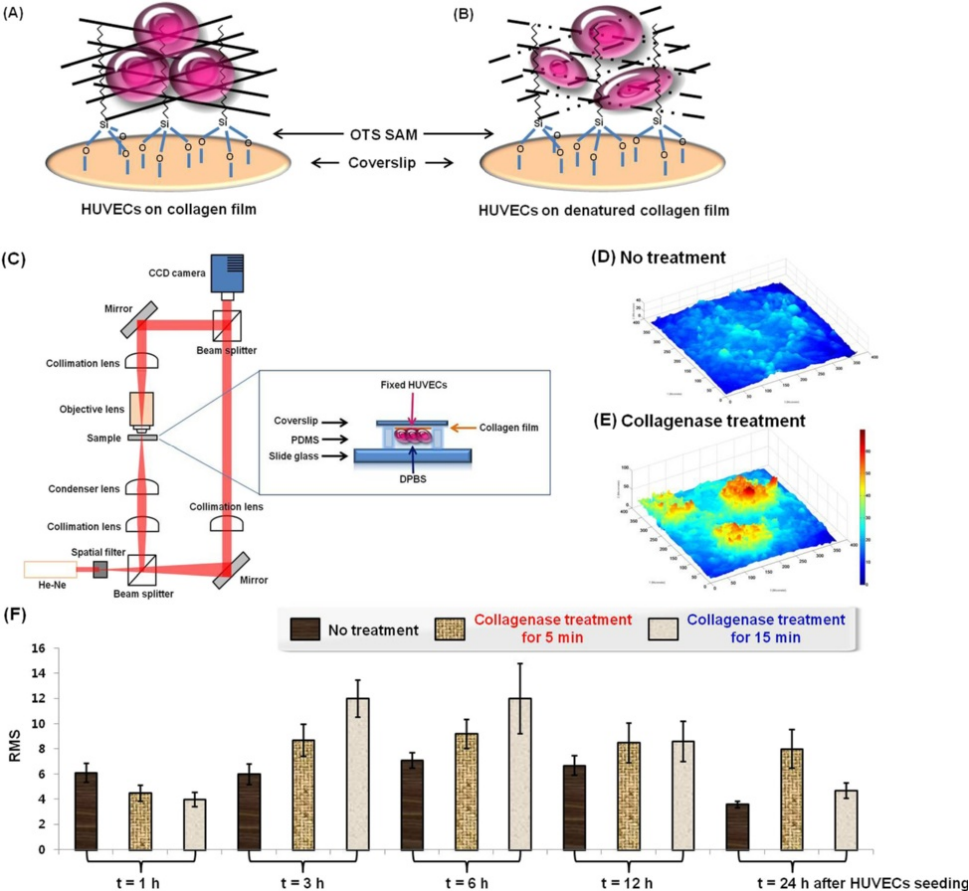
**Surface roughness of HUVECs cultured on the two collagen films.** HUVECs were incubated on top of the collagen film **(A)** and denatured collagen film **(B)**. Surface roughness of HUVECs on the two collagen films was analyzed using phase-contrast DHM. **(C)** Experimental setup of the phase-contrast DHM and cell-substrate samples used in this study. **(D and E)** Spatial distribution of physical thickness of HUVECs on the normal collagen film (non-treatment group) and denatured collagen film (treatment group). Holograms exhibit the thickness distribution of HUVECs in the non-treatment group **(D)** and treatment group **(E)**. **(F)** Temporal variations in surface roughness of HUVECs cultured on the collagen film and collagenase-treated collagen film. RMS = root mean square. *N* = 10.

The spatiotemporal thickness profiles of HUVECs cultured on collagen film (Figure 2A) or denatured collagen film (Figure 2B) were quantitatively analyzed. The biophysical properties of HUVECs grown on normal or enzyme-degraded collagen films were measured using phase-contrast DHM (Figure 2C). The typical phase-contrast DHM images in Figures 2D and 2E show the spatial distributions of the physical thickness of HUVECs cultured on collagen and denatured collagen films, respectively. We evaluated the surface roughness of HUVECs using phase-contrast DHM images. The surface roughness of ECs was represented mathematically using the root mean square (RMS) value of its fluctuations. The HUVECs initially seeded on the collagen film and denatured collagen film exhibited relatively low RMS values. Figure 2F shows that the RMS value of HUVECs cultured on collagen film slightly increased after 6 h, and then rapidly decreased in the time period (*t*) from 6–24 h. This result was attributed to normal cell adhesion to the ECM substrate. The average RMS value was approximately 3.6 μm (S.D. = 0.8) 24 h after initial seeding. This value corresponded to an approximate reduction of 59% from the initial RMS value (6.1 ± 2.3). The surface roughness of HUVECs cultured on collagenase-treated collagen film for 5 min gradually increased until *t* = 24 h after HUVEC seeding. The RMS value increased by approximately 178% at *t* = 24 h compared with the initial value (4.5 ± 1.9) at *t* = 1 h. The surface roughness of HUVECs cultured on collagenase-treated collagen film for 15 min increased more rapidly at *t* = 6 h compared with those treated with collagenase for 5 min, and then gradually decreased as the cells detached from the ECM substrate after 6 h. The RMS value increased to 300% at *t* = 3 h compared with the initial value (4.0 ± 1.7) at *t* = 1 h, and decreased by approximately 255% at *t* = 24 h. The RMS value of HUVECs cultured on normal collagen films decreased after 24 h of seeding because the cells adhered to the ECM substrate. By contrast, the RMS values of HUVECs cultured on collagen films treated with collagenase for 5 min increased because of abnormal cell deformation. The surface roughness of HUVECs on collagen films treated with collagenase for 15 min increased more rapidly than those treated for 5 min. However, the RMS value decreased after 12 h because the abnormally clustered cells were detached from the ECM substrate.

### Cell migration

**Figure 3:**
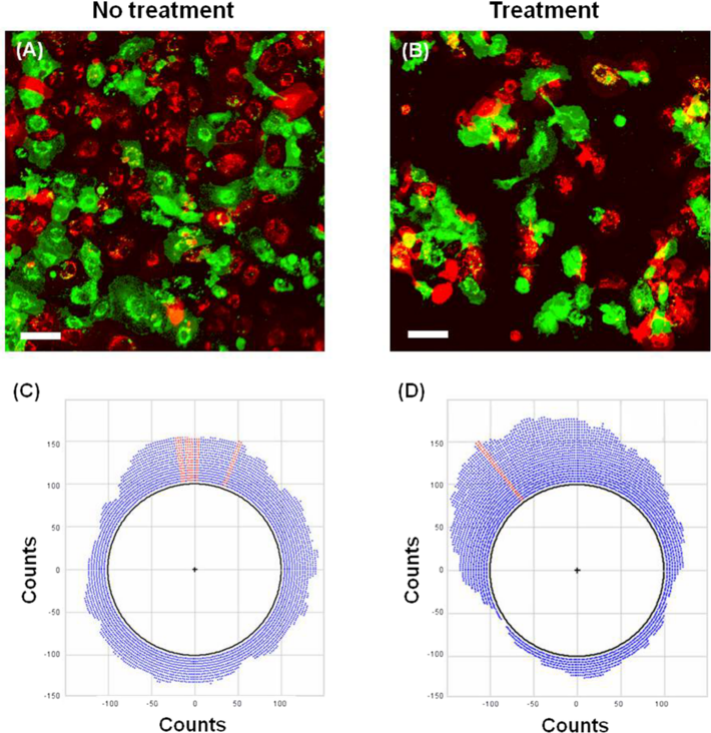
**Confocal images of HUVECs cultured on the two collagen films. (A)** Confocal images of HUVECs cultured on the collagen film (non-treatment group). **(B)** Confocal images of HUVECs cultured on the collagenase-treated collagen film (treatment group). Circular plots of the migration angle and number of HUVECs for the non-treatment group **(C)** and treatment group **(D)**. Scale bar = 100 μm.

We established two different collagen films as ECM models: the collagen film, which had a fiber structure similar to that of the ECM of normal blood vessels, and the denatured collagen film, which mimicked the ECM of atherosclerotic blood vessels. To observe the dynamic behaviors of cell-to-cell interactions, we cultured HUVECs on the collagen and denatured collagen films. Dynamic motions of ECs were observed using confocal laser scanning microscopy (CLSM). ECs cultured on collagen film or denatured collagen film were mounted in the flow chamber. Figures 3A and 3B show that ECs on the normal collagen film (non-treatment group) formed a monolayer, whereas those found on the denatured collagen films (treatment group) were aggregated and multistacked. Their movements were detected for 90 min at an interval of 10 min using CLSM. For the time-resolved tracking of ECs, cell movement was systematically analyzed by tracking the nuclei of cells. We marked the center of the nucleus of each cell to track its trajectory. The circular plots shown in Figures 3C and 3D represent the direction angle of cell migration and number of distributed cells on the collagen film and denatured collagen film. All cells were positioned at the zero point of the starting time, but these cells spread out as time progressed. As shown in the circular plots, ECs on the collagen films moved radially outward in all directions almost evenly. However, cells on the denatured collagen film moved in a preferential direction. Therefore, the non-treatment group showed a monolayer, whereas the treatment group exhibited aggregated ECs.

### Growth properties of ECs

**Figure 4:**
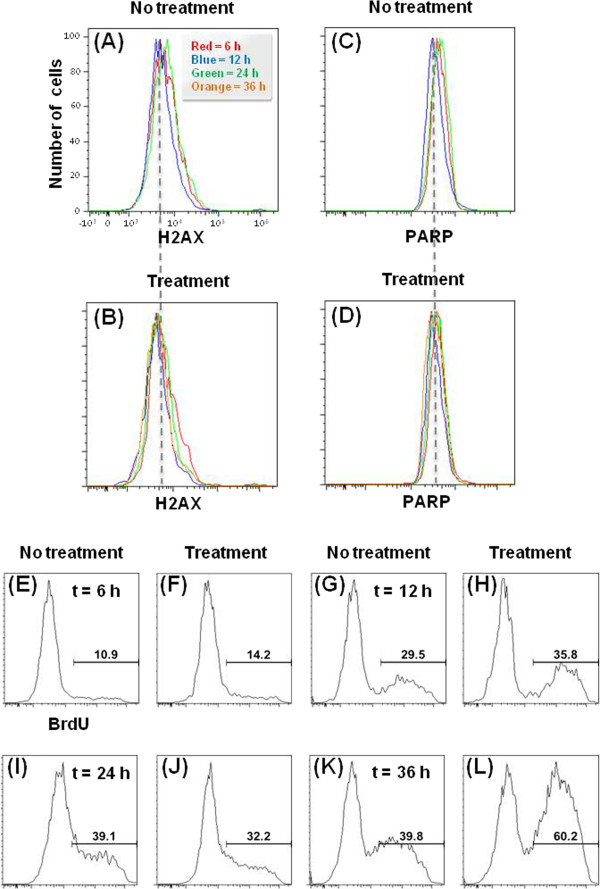
**Survival and proliferation of HUVECs.** HUVECs were cultured on both films for 6, 12, 24, and 36 h. DNA damage, apoptosis, and cell proliferation of HUVECs plated on the collagen-treated film were analyzed using flow cytometry to investigate their survival and proliferation. Comparison of histograms of H2AX-negative cells [**(A)** and **(B)**, non-DNA damage] and PARP-negative cells [**(C)** and **(D)**, non-apoptosis] on the normal collagen film (non-treatment group) and denatured collagen film (treatment group). Cell death by DNA damage (positive population of H2AX) and apoptosis (positive population of PARP) was not detected. **(E–L)** Comparison of HUVECs on the collagenase-treated collagen film showed enhanced proliferation rates.

To compare the proliferation and survival of HUVECs between the two groups, DNA damage, apoptosis, and cell proliferation were analyzed using fluorescence-activated cell sorting (FACS) technique. The control group (HUVECs on the collagen film) and treatment group (HUVECs on the denatured collagen film treated with collagenase for 5 min) were cultured for 6, 12, 24, and 36 h. As shown in Figures 3A–3D, cells with DNA damage (H2AX-positive population) and apoptosis (PARP-positive population) were not observed for both groups. H2AX-negative cells (Figures 4A and 4B) and PARP-negative cells (Figures 4C and 4D) did not show noticeable differences on both films. Furthermore, HUVECs on the denatured collagen film enhanced the proliferation rate compared with the control group (Figures 4E–4L). These results show that the aggregated form of HUVECs on the denatured collagen film was caused by the selective migration of HUVECs into a specific area or by enhanced proliferation of HUVECs in a specific area, such as the remaining collagen fibers in the denatured collagen film.

### MMP-1 and MMP-2 of ECs

**Figure 5:**
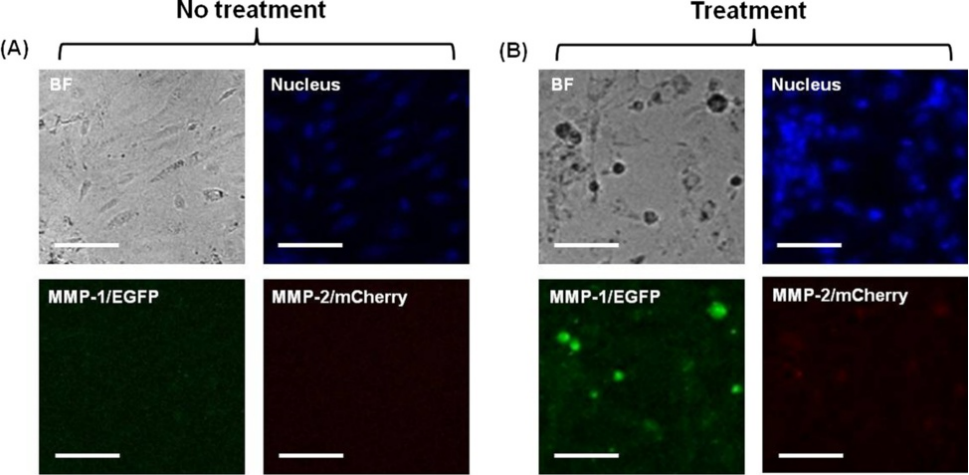
**MMP-1 and MMP-2 of HUVECs.** Bright field (gray), nucleus (blue), MMP-1/EGFP (green), and MMP-2/mCherry (red) images of normal **(A)** and abnormal HUVECs **(B)** cultured for 24 h. Scale bar = 100 μm.

HUVECs were transfected with MMP-1/enhanced green fluorescent protein (EGFP) and MMP-2/mCherry vectors to visualize the secretion of MMP-1 and MMP-2. Cells were cultured on normal (non-treatment group) and denatured collagen films (treatment group) for 24 h. These cells were then fixed with paraformaldehyde solution, and stained with 4’,6-diamidino-2-phenylindole (DAPI, blue). Figure 5A shows that MMP-1 and MMP-2 were not expressed in the non-treatment group. As shown in Figure 5B, MMP-1 and MMP-2 were observed in the treatment group. These results indicate that ECs on the denatured collagen film could secrete MMP-1 and MMP-2.

### Collagen zymography

**Figure 6:**
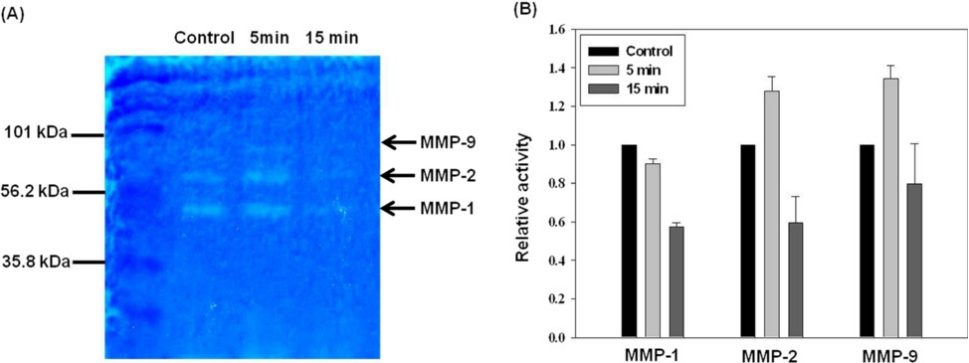
**Comparison of the activities of MMP-1, -2, and -9 in HUVECs.** MMP activity measured by collagen zymography for HUVECs cultured for 24 h. **(A)** Collagen zymography. **(B)** Densitometric analysis of MMP-1, -2, and -9 activity in HUVECs. Control, HUVECs on the collagen film; 5 min, HUVECs on the denatured collagen film treated with collagenase for 5 min; 15 min, HUVECs on the denatured collagen film treated with collagenase for 15 min. *N* = 3.

MMP is secreted as a pro-enzyme, and only activated MMP can degrade the collagen matrix. Therefore, immunofluorescent images do not show MMP activity. To overcome this limitation, we performed substrate activity using collagen zymography assay. As shown in Figure 6A, collagen zymography showed three distinct MMP bands. The lower band was MMP-1, which was about 40–60 kDa. The middle band was gelatinase-A (MMP-2), which was about 72 kDa. The upper band represented gelatinase-B (MMP-9), which was at about 95 kDa. The MMP-1 level decreased in the 5 min group compared with that in the control group (Figure 6B), and further decreased in the 15 min group. By contrast, the levels of MMP-2 and MMP-9 increased in the 5 min group compared with those in the control group. However, they significantly decreased in the 15 min group.

### Expression of E-selectin, VCAM, CD146, and VE-cadherin

**Figure 7:**
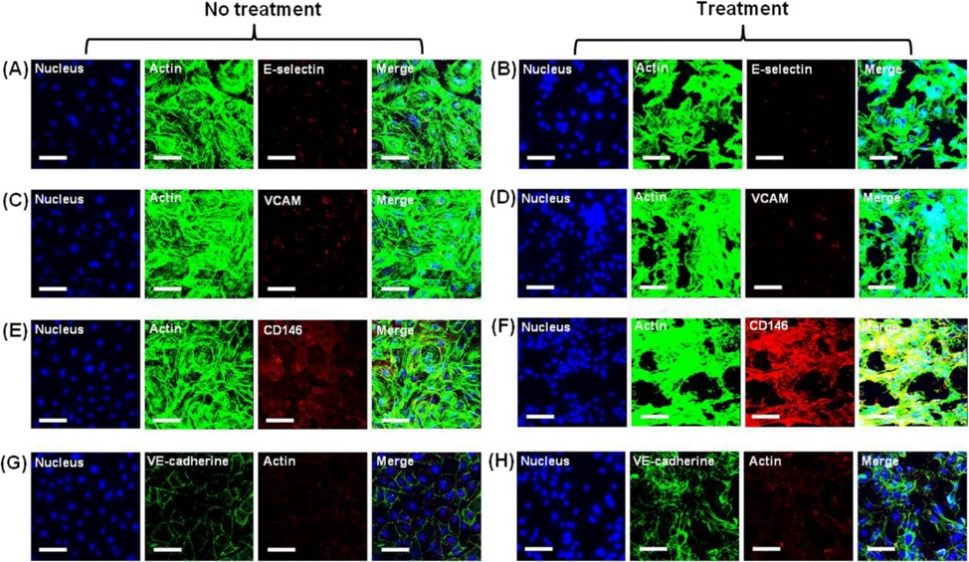
**Comparison of the expression of E-selectin, VCAM, CD146, and VE-cadherin.** HUVECs cultured for 24 h on the collagen film **(A, C, E, and G)** and denatured collagen film treated with collagenase for 5 min **(B, D, F, and H)**. Fluorescence images of actin [red in **(G)** and **(H)**, green in other figures], nucleus (blue), adhesion molecules (green in VE-cadherin and red in E-selectin, VCAM, and CD146), and their merged images. E-selectin **(A, B)**, VCAM **(C, D)**, CD146 **(E, F)**, and VE-cadherin **(G, H)**. Scale bar = 250 μm.

The expression levels of E-selectin, VCAM, CD146, and VE-cadherin on the EC surface for the two collagen films were visualized using immunostaining method and confocal imaging technique. Among the four adhesion molecules, CD146 and VE-cadherin were expressed on the EC surface cultured on both collagen films (Figure 7). Figure 7F shows that CD146 expression significantly increased on the EC surface of the denatured collagen film compared with that on ECs cultured on normal collagen film (Figure 7E). The results from the present *in vitro* studies on the cultured ECs suggest the important function of the biophysical properties of ECM in the formation of the EC layer. In particular, MMP-1 and MMP-2 caused weak cell interactions. Therefore, CD146 expression increased on the EC surface of the denatured collagen film.

### Bead accumulation on the EC layer

**Figure 8:**
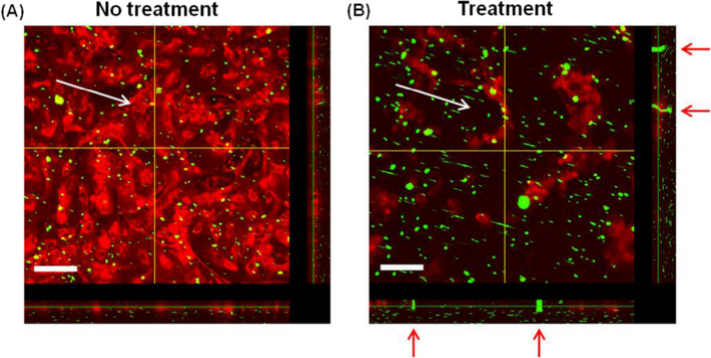
**Confocal images of beads accumulated on the HUVEC layers. (A)** Accumulated beads (green) on the normal HUVEC layer (non-treatment group). **(B)** Accumulated beads (green) on the abnormal HUVEC layer (treatment group). The images were captured after 1 min under a flow rate of 1 mL/h (shear stress: 23.96 mPa). White arrows indicate the direction of medium flow, and red arrows in **(B)** denote the bead clusters accumulated near the HUVEC clusters. The cross-sectional profiles are displayed along the yellow lines in the right ends and bottom parts. Scale bar = 100 μm.

**Figure 9:**
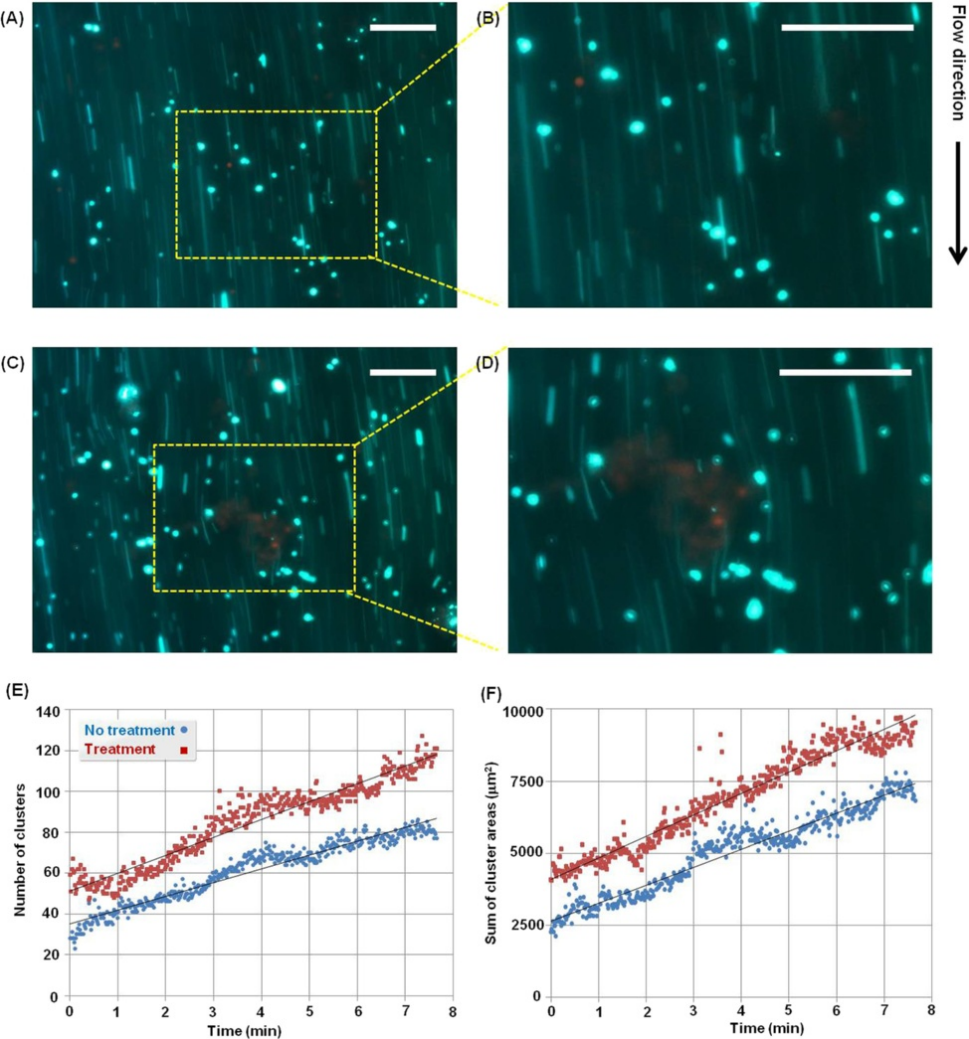
**Bead accumulation on HUVECs cultured on the two collagen films.** Fluorescent images of accumulated beads (green) on the normal **(A and B)** and abnormal HUVEC (red) layers **(C and D)**. The images were captured after 1 min under a flow rate of 1 mL/h (shear stress: 23.96 mPa). The images in the yellow dotted box were magnified two times **(B and D)**. Scale bar: 100 μm. **(E and F)** Temporal variations in the number of clusters **(E)** and sum of cluster areas **(F)**.

ECs cultured on the collagen film or denatured collagenase-treated collagen film for 24 h were mounted on the flow chamber. The dynamic motion of beads on the cultured ECs was observed by time-lapse microscopy. Weakly adhered beads were detached under flow conditions, and some beads attached to the HUVEC layer were continuously concentrated to form EC clusters. In HUVECs cultured on the collagen film and denatured collagen film for 24 h, the size and number of clusters gradually increased (data not shown) as the flow rate increased. The beads were clustered on the HUVEC layers, and the cluster size increased. Figures 8A and 8B show the confocal images captured after 1 min at a flow rate of 1 mL/h. Figures 9A–9D show the fluorescence images captured after 1 min at the same flow rate. As shown in these figures, the beads on the normal EC layer (Figures 6A, 7B, and 9A) were less accumulated than those on the abnormal EC layers (Figures 8B, 9C, and 9D). In addition, the clusters in the treatment group were larger than those in the non-treatment group. Figures 9E and 9F show the temporal variations in the number of clusters and in the sum of cluster areas, respectively. These values were obtained from time-lapse images recorded consecutively at a flow rate of 1 mL/h. In Figure 9E, the number of clusters gradually increased, and the number of clusters on the normal EC layer was lower than that on the abnormal EC layer. Figure 9F shows that the beads on the normal EC layer were less accumulated than those on the abnormal EC layer. The slope increased by approximately 30% for the abnormal EC layer group compared with that for the normal EC layer group. In addition, the sum of cluster area in the collagenase-treated group was higher than that of the non-treatment normal group. The slope was higher (18.8%) for the abnormal EC layer group than that for the normal EC layer group.

## Discussion

In this study, the expression levels of MMP-1 and MMP-2 were observed in ECs on denatured collagen film. The results show that the destruction of the ECM structure was caused by MMP-1 and MMP-2, which was secreted by ECs on denatured collagen film. In addition, MMP-1 and MMP-2 could influence the formation of EC clusters and detachment of EC clusters from the EC layer. The experimental results obtained from the present *in vitro* studies on the cultured ECs suggest that the biophysical properties of ECM had an important function in the formation of the EC layer. Only HUVECs grown on the denatured collagen film expressed MMP-1 and MMP-2, whereas no MMP expression was found in HUVECs grown on the collagen film. Previous studies have shown that HUVECs grown on collagen gels express MMP-1, -2, and MT1-MMP to degrade the collagen matrix [49,50]. Consequently, MMP expression is higher on stiffer matrix (collagen film) compared with that on softer matrix (denatured collagen film) [51,52]. Given that MMP is secreted as a pro-enzyme (pro-MMP) and only activated MMP can degrade the collagen matrix, immunofluorescent images do not show much about MMP activity [53,54]. The collagen zymography data obtained in this study show that HUVECs on the three different collagen films had relatively different MMP activities. Zymography is an electrophoretic technique to detect hydrolytic enzymes based on the substrate repertoire for the enzyme. Collagen zymography allows us to examine MMP activity, whereas fluorescence images of MMP in Figure 5 distinguish whether the MMP is present or not. Thus, the fluorescence images showing the expression of MMPs in Figure 5 are different from the zymography data showing the activity of MMPs in Figure 6.

HUVECs adhered to the collagen films during collagen zymography, but the experimental results do not reflect the entire endothelium. To overcome this limitation, further collagen zymography experiments using detached ECs (circulating ECs) or MMPs secreted by ECs are required in the future.

All the experiments were performed by culturing ECs on the collagen films for up to 24 h. At 24 h after seeding of ECs, HUVECs clustered on the denatured collagen films gradually detached from the substrate. Given the difficulty in quantitatively analyzing the cells because of their complexity, we only carried out the experiments for up to 24 h.

Among several adhesion molecules, CD146 expression significantly increased on the EC surface of the denatured collagen film compared with that on ECs cultured on normal collagen film. The adhesion molecules of ECs possibly modulated gene expression by changing the morphological structures of the ECM by MMP-1 and MMP-2. These molecules may activate many signaling molecules in focal adhesion and cytoplasm by dynamically interacting with ECM proteins. The denatured ECM may cause endothelial dysfunction by mechanotransduction mechanisms. CD146 is a marker for circulating endothelial cells. The increased levels of CD146 are positively correlated with active inflammatory reactions in idiopathic myopathy [55], chronically inflamed tissues [56], inflammatory skin disease [57], rheumatoid arthritis [58], inflammatory bowel disease [59,60], chronic obstructive pulmonary disease [61], and multiple sclerosis disease [62,63]. The engagement of CD146 initiates the protein kinase phosphorylation cascade through association with Fyn, a Src family kinase. Phosphorylated Fyn subsequently transfers phosphate to the downstream kinase of PKC-γ, which triggers a Ca^2+^ burst within cells. Consequently, the induced association among proteins of P130, Pyk2, and paxillin, as well as activated p125 (FAK), promotes the polarization actions of actins. Thus, this CD146-mediated signaling pathway can be used to decipher the mechanism that CD146 promotes cell motility through extracellular signals to downstream-signaling components for cytoskeleton remodeling [64,65]. The integrins of ECs may activate many signaling molecules in focal adhesion and cytoplasm via dynamic interaction with collagen fibers. Therefore, the mechanical properties of denatured collagen fibers may contribute to the preferential EC migration on the denatured collage film. For better understanding of its function in signaling transduction, further study on the crosstalk with members of various signaling pathways is required [66].

The functional assessment of ECs can be used to identify endothelial damage and predict cardiovascular risks. However, this type of assessment does not provide sufficient information about the mechanisms underlying the development of endothelial dysfunction [67,68].

In this study, the flow rates used for bead accumulation experiment do not fully reflect the *in vivo* pathophysiological conditions. To select effective drugs for circulatory vascular diseases, the size and surface properties of the test drugs and flow condition (flow rate or low endothelial shear stress) of microfluidic devices are highly important. In particular, the physical dimensions of the microfluidic devices used in the present *in vitro* study should be optimized based on data obtained from *in vivo* disease models to maximize the throughput.

## Conclusions

Using an *in vitro* disease model, we observed the endothelial dysfunction, such as disorganization and thickening, of the EC layer. HUVECs cultured on normal collagen films were thinly adhered to the substrate. By comparison, HUVECs cultured on denatured collagen films exhibited abnormal cell growth, such as aggregation and cluster formation of ECs. The expression levels of MMP-1, MMP-2, and CD146 increased in ECs on the denatured collagen film. These results indicate that the denatured ECM was closely related to endothelial dysfunction, which is one of the main pathogeneses of circulatory vascular diseases. The morphological structures of the denatured collagen film likely caused EC deformation. In addition, more fluorescent beads accumulated on the EC layer on the denatured collagen film. These *in vitro* studies would be useful for understanding the outbreak mechanism of circulatory vascular disorders. *In vitro* studies using a bio-inspired ECM could contribute in developing a unique experimental modality for optimizing the design parameters to screen effective therapeutic drugs, and determine the physical dimensions and flow rate condition of a microfluidic device used for clinical treatments of circulatory diseases by matching *in vivo* pathophysiological conditions.

## Methods

### Denatured collagen film

The substrates of 18 mm round cover glasses (Marlenfeld GmbH & Co. KG, Lauda-Königshofen, Germany) were coated with silicon nitride (coating thickness = 200 nm). The cover slips were then treated with reactive O_2_ plasma (20 sccm, 200 mTorr, 50 W for 3 min) to simulate the morphological structures of normal collagen films. A methyl-terminated SAM was created at room temperature by incubating the substrate overnight with 3.78 μL of OTS mixed with 10 mL of anhydrous toluene solution. The samples were washed with toluene solution, and baked in an oven at 90°C for 20 min. The substrate was cleaned by sonication in toluene solution for 1 min, washed with 70% ethanol, and dried under nitrogen gas stream. To prepare the collagen films on a glass surface, we diluted 1 mg/mL collagen type I (from rat tails; Invitrogen, Carlsbad, CA, USA) to a final concentration of 0.32 mg/mL using PBS (pH 7.4) solution, which contained calcium and magnesium; the resulting mixture was incubated overnight with previously prepared methyl-terminated glass at 37°C [69]. The samples were then washed with PBS solution (without calcium and magnesium). Collagenase type I (from *Clostridium histolyticum*; Sigma-Aldrich) was diluted to a final concentration of 2 mg/mL using PBS solution containing calcium and magnesium. The prepared collagen film surface was treated for 5 or 15 min to form denatured collagen films on a glass surface. The collagen and denatured collagen-conjugated glass surfaces were immediately washed with PBS solution (without calcium and magnesium). For SEM imaging, both collagen films were rinsed with deionized water and dried with nitrogen gas for the analysis of structural properties. The samples were mounted on metal stubs and coated with platinum (SC7640 model, Quorum Technology). SEM images were captured using SEM (JEOL JSM-7401 F, Japan) at an acceleration voltage of 15 kV. Surface structures of the two films were also observed by AFM (VECO Dimension 3100). The three-dimensional (3D) reconstruction of AFM images was performed using Nanoscope V (Version 7.0) software. Based on a previous study by Elliot et al., the collagen thin film coated on the OTS-SAM-coated glass is amenable for use in engineering applications and for manipulating and studying cell behaviors. This reproducible and analytically tractable collagen matrix provides a unique tool for the systematical examination of matrix organization and composition [69].

### Cell preparation

All experimental procedures complied with the act on life ethics and safety of the Ministry of Health and Welfare of South Korea. HUVECs were obtained from Invitrogen (Carlsbad, CA, USA) and used in passages 5 or 7. The cells were cultured in Medium 200 (Gibco) with low-serum growth supplement (Gibco) containing 20% fetal bovine serum (Gibco) and 1% penicillin-streptomycin (HyClone). The cells were cultured in a T75 flask (Falcon) under a humidified atmosphere of 5% CO_2_ at 37°C. Trypsin/ethylenediaminetetraacetic acid solution was used to detach cells from the culture flask. The surface-treated cover glass (collagen film or denatured collagen film) was placed in a cell culture dish (diameter = 35 mm), loaded with 10^6^ cells/mL cell suspension, and incubated under a humidified 5% CO_2_ atmosphere at 37°C. Figures 1A and 1B illustrate the ECs cultured on the collagen and denatured collagen films, respectively. HUVECs were cultured on the collagen film or denatured collagenase-treated collagen film for 1, 3, 6, 12, and 24 h. For DHM imaging, the test samples were fixed with 4% paraformaldehyde solution for 20 min at room temperature, and washed with PBS solution. Each sample was placed on a drop of PBS solution at the center of a polydimethylsiloxane chamber. Sample images were photographed at room temperature.

### Phase-contrast holographic microscopy

He-Ne (λ = 633 nm) laser was used as a light source of an upright microscope (Eclipse i50, Nikon) equipped with a 20× objective lens (NA = 0.5). Using additional relay optics, we set the overall magnification of the microscope at approximately 30×. Interferograms were recorded using a charge-coupled device (CCD) camera (PCO 2000, 7.4 μm/pixel) of 2 k × 2 k pixel resolution. The transmission-type phase-contrast DHM applied in this study is based on the principle of the Mach-Zehnder interferometer. After passing through a spatial filter, a laser beam was divided into an object beam and reference beam by a beam splitter. The sample was illuminated by the object beam via a condenser lens. A microscope objective (MO) lens caused the wavefront in the object beam to form a curved line, which resulted in the deformation of the object wave phase. The objective beam was collimated using a collimation lens to compensate the phase aberrations caused by MO. The collimated object wave interfered with the collimated reference wave at a slightly tilted incidence angle to generate holograms.

### Morphology and surface roughness of EC layer

An angular spectrum algorithm was employed to reconstruct holograms [43,70,71]. This technique does not require a minimum distance between the object plane and hologram plane. The angular spectrum algorithm is flexible and can be effectively used to filter the frequency domain with high accuracy. If *E*_0_(*x*_0_, *y*_0_; 0) is the wave field at plane *z* = 0, the angular spectrum *A*(*k*_*x*_, *k*_*y*_; 0) of the hologram at *z* = 0 is obtained by Fourier transformation:

(1) $$A\left(k_{x},k_{y};0\right)=\int \int E_{0}\left(x_{0},y_{0};0\right)\text{exp}[-i(k_{x}x_{0}+k_{y}y_{0})]dx_{0}dy_{0}$$

where *k*_*x*_ and *k*_*y*_ are the spatial frequencies of the *x* and *y* components, respectively.

The separated zero-order image, virtual image, and real image were clearly observed in the frequency domain. Fourier-domain filtering was applied to the angular spectrum to eliminate unwanted noises, as well as the zero-order and virtual images. A region of interest in the real image was selected using Fourier-domain filtering. The actual image was shifted to the center of the frequency domain. The complex wave field, reconstructed at any plane perpendicular to the propagating *z-*axis, was then calculated using the following equation:

(2) $$\begin{array}{ll} E\left(x,y;z\right) & ={ℑ}^{-1}\left\{\text{filter}\left[ℑ\left\{E_{0}\right\}\right]\text{exp}\left[ik_{z}z\right]\right\},\\ \;k_{z} & =\sqrt{k^{2}-{k_{x}}^{2}-{k_{y}}^{2}} \end{array}$$

where *filter*[*ℑ*{*E*_0_}] denotes the filtered angular spectrum, and *ℑ* and *ℑ*^- 1^ are the Fourier transform and inverse Fourier transform, respectively. The reconstructed wave field *E(x*,*y*;*z)* is represented by an array of complex numbers. The amplitude-contrast images were obtained by calculating the intensity distribution as follows:

(3) Im,n=ReEx,y;z2+ImEx,y;z2

and the phase-contrast image was derived from the following argument values:

(4) ϕm,n=arctanImEx,y;zReEx,yl;z

The 2π ambiguity encountered in the wrapped phase image was resolved by the unwrapping process [72]. The physical thickness of cells was calculated using the following equation:

(5) $$d=\lambda \left(\phi /2\pi \right)/\left(n-n_{0}\right)$$

where λ is the wavelength, *ϕ* is the unwrapped phase angle, and (*n-n*_*0*_) is the refractive index difference between the cells and surrounding medium.

The hologram images of the field-of-view of approximately 370 μm × 370 μm were captured using a CCD camera with 1600 × 1600 pixels. Considering that the refractive index depends on principal components and different physical states, we discovered that the local refractive index was slightly different in the EC layer. The mean refractive index of ECs tested in this study was approximately 1.375. The stability of the mean refractive index corresponds to the vertical sensitivity of approximately 11 nm [42,73,74]. Assuming that the total refractive index of the cells is constant, we applied Equation (5) to extract information about physical thickness from the original phase image. The obtained 3D phase image clearly shows the physical thickness distribution of the EC layer with high spatial resolution.The 3D phase images shown in Figures 2D and 2E illustrate the physical thickness distribution of the EC layer with high spatial resolution. Variations in the surface roughness of HUVECs were described mathematically using root mean square (RMS). The surface roughness of cells was calculated using the following equation:

(6) $$x_{\text{rms}}=\sqrt{\frac{1}{n}\sum M^{2}}$$

where *n* is the number of samples, and *M* is the thickness of the EC layer.

### Dynamic behavior of ECs

To observe the dynamic behavior of cell-to-cell interaction, we visualized HUVECs cultured on collagen or collagenase-treated collagen film using CLSM (OLYMPUS FV-1000). HUVECs’ membranes were labeled with a PKH26 red fluorescent cell linker kit (Sigma-Aldrich) and PKH67 green fluorescent cell linker kit (Sigma-Aldrich) according to the manufacturer’s protocol.

The green- and red-fluorescence-labeled HUVECs were mixed at a volume ratio of 1:1. These cells were seeded on collagen film, and incubated under humidified 5% CO_2_ medium at 37°C. To maintain the standard tissue culture condition for ECs in the flow chamber, we placed all the components, except the syringe pump, in a chamber (Chamlide TC, Live Cell Instrument, Korea) in which the temperature and CO_2_ concentration were controlled. The cell culture medium was continuously circulated (1 mL/h) over the cells by a syringe pump (KD Scientific). ECs were cultured on the collagen film or denatured collagenase-treated collagen film for 3 h, and mounted in the flow chamber. Cell movements were observed for 90 min at an interval of 10 min under CLSM.

### Growth properties of ECs

The growth properties of HUVECs plated on the treated and untreated collagen films were analyzed using the Apoptosis, DNA damage, and Cell Proliferation (ADDCP) kit (BD Biosciences, USA) and FACS technique. The ADDCP kit contains key markers [bromodeoxyuridine (BrdU), H2AX, and PARP] for the simultaneous determination of important cellular state changes in the cell cycle, such as apoptosis, DNA damage, and cell proliferation. BrdU is an analog of the DNA precursor thymidine. When cells are incubated in the presence of BrdU, the molecule is incorporated into newly synthesized DNA and can be detected with antibodies against BrdU [75]. DNA damage was determined using Phosphorylated H2AX. H2AX is a member of the histone H2A protein family. Phosphorylation of H2AX leads to the recruitment of DNA damage repair proteins at the site of DNA damage [76]. Detection of cleaved PARP is used for the study of apoptosis. During the early phases of apoptosis (programmed cell death), caspase-3 is activated by cleavage [77].

HUVECs were cultured on the collagen film or denatured collagen film for 6, 12, 24, and 36 h. Biological analyses on apoptosis, DNA damage, and cell proliferation were performed according to the manufacturer’s protocol. The test samples were then analyzed using a BD FACSCanto II flow cytometer (BD Biosciences, USA). The acquired data were handled with FlowJo (Version 10.0.6) flow cytometry analysis software.

### MMP-1 and MMP-2 of ECs

MMP-1 (BC013875.2) and MMP-2 (BC002576.2) in the gateway entry vector were tagged with EGFP or red fluorescent protein (mCherry) by combining the MMPs with gateway destination vector (pDS-XB-EGFP or pDS-XB-mCherry) using LR recombinase (Invitrogen). MMP-1 or MMP-2 vector was mixed with FuGENE HD transfection reagent (Promega). HUVECs were then tagged with MMP-1/EGFP and MMP-2/mCherry. To visualize the spatial distribution of MMP-1 or MMP-2 vector on HUVECs, we cultured HUVECs on collagen or denatured collagenase-treated collagen films for 24 h, and fixed the cells with 4% paraformaldehyde solution for 20 min at room temperature. ECs were then stained with DAPI (Invitrogen) for nuclear staining. The stained EC images were obtained using CLSM (TCS SP5II MP Leica Microscopy Systems, GMBH) with a 20× IR APO water-immersion lens (NA 1, Leica Microscopy Systems, GMBH). The acquired images were analyzed and processed using LAS AF 2.7 software (Leica Microscopy Systems, GMBH). MMP2 has difficulty in discriminating the difference in fluorescence intensity of ECs on the collagen and denatured collagen films because of their weak fluorescence intensities. Therefore, we further improved the contrast and brightness of fluorescence images using Image J software.

### Collagen zymography

Collagen zymography assay was performed to detect MMP-1, -2, -9, and -13 [78,79]. To investigate MMP activity on the three different collagen films, samples were centrifuged to dispose cellular debris and then homogenized without a reducing agent to retain the native state of the enzyme. Samples were centrifuged once again at 16,000 g for 20 min at 4°C. The protein concentration of the collected supernatant was measured by a Nanodrop Spectrophotometer (Thermo Scientific, Wilmington, DE, USA). The samples were mixed with an equal volume of 2× non-reducing sample buffer (Santa Cruz Technology, Santa Cruz, CA, USA), and 15–20 μL was loaded in each well. Polyacrylamide gels containing 10 mL of type I collagen (from rat tails; 1 mg/mL, Invitrogen, Carlsbad, CA, USA) were dispersed in a buffered solution consisting of 2.5 mL of gel, 1.5 M Tris–HCl (pH 8.8), 165 μL of 10% SDS, 5.25 mL of 40% polyacrylamide, 165 μL of 50% glycerol, and 4 mL of distilled water. The stacking gel containing 4% polyacrylamide in 1 M Tris–HCl (pH 6.8) was polymerized by adding 100 μL of 10% ammonium persulfate and 10 μL of TEMED. The gel was then electrophoresed at a constant voltage of 90 V. The electrophoresed gel was washed twice in 200 mL of 2.5% Triton X-100 (30 min each) under shaking, and incubated in 100 mM Tris–HCl, 5 mM CaCl_2_, 0.005% Brij-35, and 0.001% NaN_3_ (pH 8.0) for 6–48 h at 37°C. The gel was stained with 0.25% Coomassie brilliant blue G-250 (50% methanol, 10% acetic acid) for 1 h at room temperature and destained (40% methanol, 10% acetic acid). The gels were incubated for 1 h in 5% methanol and 7.5% acetic acid, and kept under cellophane at 4°C. The stained gel images were obtained using a color scanner. The density of MMP bands in the acquired images was analyzed using Quantity One software (Bio-Rad Laboratories, Hercules, CA, USA).

### Immunostaining of ECs

HUVECs were cultured on collagen film or denatured collagenase-treated collagen film for 24 h, and fixed with 4% paraformaldehyde solution for 20 min at room temperature to visualize the spatial distribution of adhesion molecules on HUVECs. The antibodies (5 μg/mL) for VCAM, E-selectin, CD146, and VE-cadherin (R&D Systems, USA) were overlaid for 1 h on the EC surface and washed. The secondary antibody (10 μg/mL) conjugated with Alexa Fluor 488 or 594 (Invitrogen) was added again for 1 h and rinsed. ECs were then stained with phalloidin tetramethylrhodamine isothiocyanate (Sigma-Aldrich) or phalloidin-fluorescein (Sigma-Aldrich) for actin staining and with DAPI (Invitrogen) for nuclear staining. The stained EC images were obtained using CLSM (TCS SP5II MP Leica Microscopy Systems, GMBH) with a 20× IR APO water-immersion lens (NA 1, Leica Microscopy Systems, GMBH). The acquired images were analyzed and processed using LAS AF 2.7 software (Leica Microscopy Systems, GMBH).

### Bead accumulation on the EC layer

A syringe pump (KDS100: KD Scientific), syringe filled with 1 mL of Medium 200 (microsphere density in medium: 9.1 × 10^7^ particles/mL) mixed with yellow-green fluorescent beads (FluoSpheres Collagen I-Labeled Microspheres, 1.0 μm), and rectangular flow chamber (Chamlide CF, Live Cell Instrument, Korea) were serially connected to silicon tubes (inner diameter/outer diameter = 1.15 mm/3.2 mm, Korea Ace) to examine the effect of flow on bead accumulation over HUVEC layers. The test channel in the flow chamber had a height, width, and length of 0.2, 2, and 14 mm, respectively. To maintain the standard tissue-culture conditions (37°C, 5% CO_2_) in the flow chamber for ECs, we placed the components, except the syringe pump, in the temperature and CO_2_ control chamber (Chamlide TC, Live Cell Instrument, Korea). The ECs cultured on collagen film or denatured collagenase-treated collagen film for 24 h were mounted in the flow chamber. Dynamic motions of the motile beads in the flow chamber were observed using CLSM (OLYMPUS FV-1000) while recording the bead accumulation process on the HUVEC layers. A modified Zeiss Axiovert 200 fluorescence microscope with a 20× (NA = 0.4) phase-contrast objective lens and AxioCam MRc CCD camera was used to monitor the temporal variation in bead accumulation on the HUVEC layers. An X-Cite 120 Q excitation light source (120 W mercury vapor short arc lamp) and shift-free filter set for GFP/RFP were used in the fluorescence imaging experiment. The microscope was operated by Axiovision 4.8.2 (Carl Zeiss). Captured images were analyzed and processed using Image-Pro Plus 7.0 software. The dynamic motions of the motile beads inside the flow chamber were observed by time-lapse microscopy for 10 min. Images were consecutively recorded at an interval of 1 s.

## Abbreviations

ADDCP: Apoptosis, DNA damage, and cell proliferation; AFM: Atomic force microscopy; BrdU: Bromodeoxyuridine; CCD: Charge-coupled device; DAPI: 4’,6-diamidino-2-phenylindole; DHM: Digital holographic microscopy; EC: Endothelial cell; ECM: Extracellular matrix; EGFP: Enhanced green fluorescent protein; CLSM: Confocal laser scanning microscopy; FACS: Fluorescence-activated cell sorting; HUVECs: Human umbilical vein endothelial cells; MMPs: Matrix-degrading metalloproteinases; MO: Microscope objective; OTS: Octadecyltrichlorosilane; RMS: Root mean square; SEM: Scanning electron microscopy; 3D: Three-dimensional; VCAM: Vascular cell adhesion molecule; VE-cadherin: Vascular endothelial cadherin.

## Competing interests

The authors declare that they have no competing interests.

## Authors’ contributions

ES: design of cell biology experiments, data collection, and data analysis; KWS: experimental set-up and analysis of DHM; JEG: cell biology experiments; YRH: collagen zymography; EY: data analysis; SL: data collection and analysis; SJL: experimental design. All authors read and approved the final manuscript.
